# Isolated endogenous *Nocardia* endophthalmitis after immunosuppression

**DOI:** 10.1007/s12348-011-0057-3

**Published:** 2012-01-26

**Authors:** Lisa Y. Chen, Muge R. Kesen, Abdalhossein Ghafourian, Quan D. Nguyen, Charles G. Eberhart, Diana V. Do

**Affiliations:** 1 The Wilmer Eye Institute, Johns Hopkins University School of Medicine, 600 North Wolfe Street, Maumenee 745, Baltimore, MD 21287 USA; 2 Department of Pathology, Johns Hopkins University School of Medicine, Baltimore, MD USA

**Keywords:** Endogenous, Endophthalmitis, *Nocardia*, Immunosuppression, Steroids

## Abstract

**Purpose:**

This study is aimed to report a case of endogenous *Nocardia* endophthalmitis in the setting of immunosuppression from chronic steroid use.

**Methods:**

A case report was conducted.

**Results:**

A 79-year-old woman presented with decreased vision with floaters in the left eye. Ophthalmic examination revealed severe inflammation in the anterior chamber, vitreous opacities, and retinal detachment. Vitreous cultures grew *Nocardia farcinica* without any systemic foci of infection found during further workup. The patient was treated with intravitreal amikacin and oral trimethoprim-sulfamethoxazole, and her retinal detachment was later repaired in the operating room. The patient has since remained stable with no signs of retinal detachment or active infection.

**Conclusions:**

*Nocardia* endophthalmitis is a rare, but serious intraocular infection that should be considered in the differential diagnosis in any immunosuppressed patient, including those receiving steroids, who presents with signs of intraocular infection.

## Introduction

*Nocardia* are aerobic, Gram-positive, weakly acid-fast, filamentous bacteria that cause opportunistic infections in immunocompromised patients. Although most commonly seen in the lungs, brain, and other soft tissues, these organisms are rare but significant causes of intraocular infection [1]. Herein, we present a case of endogenous *Nocardia* endophthalmitis in a patient with giant cell arteritis.

## Case description

A 79-year-old woman presented complaining of decreased vision with floaters in the left eye for 2 weeks. Past medical history was significant for giant cell arteritis treated with 40 mg/day oral prednisone for 3 months.

**Fig. 1:**
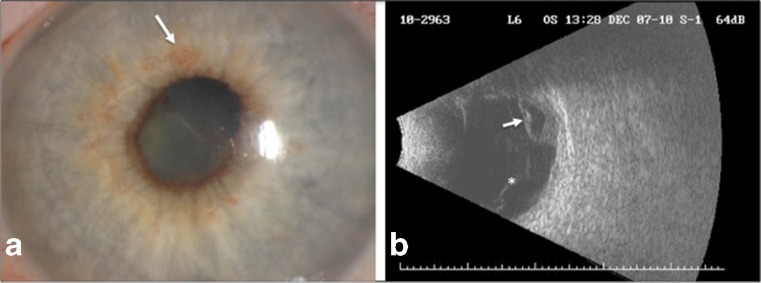
Exam findings at initial presentation. **a** Slit-lamp photograph shows inflammation in the anterior chamber with fibrin formation, poorly dilated pupil, and early rubeosis (*arrow*). **b** B-scan ultrasonography reveals vitreous opacities (*asterisk*) and partial retinal detachment with subretinal fluid (*arrow*)

Visual acuity (VA) was 20/25 in the right eye and 6/200 in the left eye. Slit-lamp examination of the right eye was unremarkable. Examination of the left eye revealed severe inflammation with fibrin in the anterior chamber, poorly dilated pupil, and early rubeosis (Fig. 1a). As there was no view of the posterior segment in the left eye, B-scan ultrasonography was performed, which showed vitreous opacities and partial retinal detachment with subretinal fluid (Fig. 1b).

The patient was started on intravenous broad-spectrum antibiotics and underwent diagnostic vitrectomy followed by intravitreal injections of vancomycin, ceftazidime, foscarnet, and amphotericin. When vitreous cultures grew *Nocardia farcinica*, the patient was admitted for further workup. During this admission, the patient remained afebrile and asymptomatic with a normal white blood cell count and negative blood cultures. Further diagnostic workup, including a CT of the chest and an MRI of the brain, did not reveal any lesions suggestive of any systemic source of infection. As such, after receiving a dose of intravitreal amikacin, the patient was discharged on oral trimethoprim-sulfamethoxazole (TMP-SMX) and advised to follow-up in clinic.

**Fig. 2:**
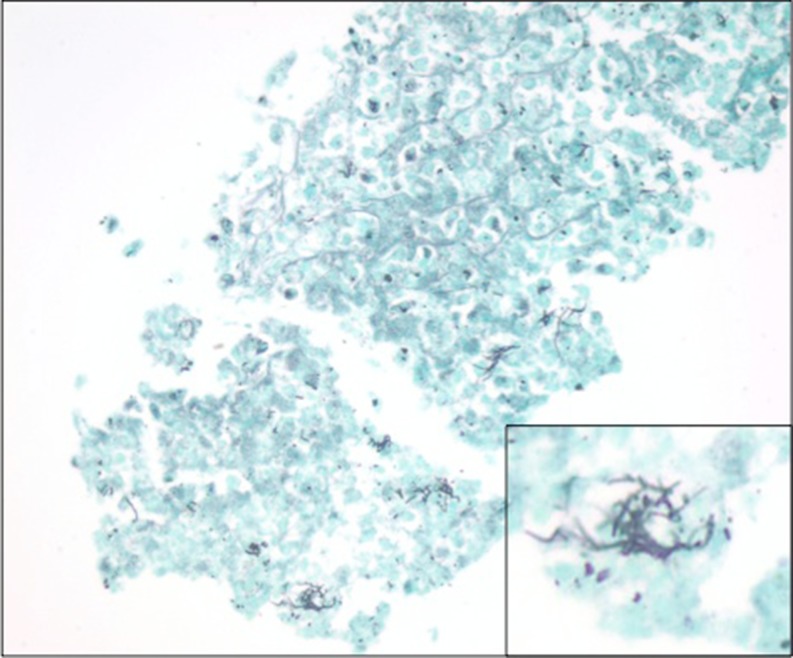
Gomori methenamine silver stain of the subretinal mass reveals numerous organisms (original magnification × 400). *Inset*: Characteristic filamentous and bacillary morphology of these organisms is consistent with *Nocardia* (original magnification × 1000)

The patient later returned to the operating room to repair the retinal detachment. Intra-operatively, the patient was noted to have a tractional retinal detachment involving the macula that spanned from the 4–8 o’clock position. A fibrous, inflammatory subretinal mass was removed inferiorly just beyond the inferotemporal arcade and sent for pathology, which revealed numerous organisms morphologically consistent with *Nocardia* on silver stain (Fig. 2). Examination 1 week postoperatively revealed an attached retina with no additional foci of infection. Since then, the patient has remained stable with VA in the left eye of E card at 4 ft at her last visit. TMP-SMX was discontinued at that time as 6 months of treatment had been completed.

## Discussion

*Nocardia* endophthalmitis is a rare, but serious intraocular infection. It is usually exogenous in origin, occurring after ocular trauma or surgery [2, 3]. Endogenous *Nocardia* endophthalmitis is more uncommon, occurring in immunocompromised patients as the result of hematogenous dissemination, usually from a primary pulmonary focus [4–6]. Occasionally, this type of endophthalmitis can occur in isolation without a systemic focus of infection; however, this is even more rare with only two reported cases in the literature [7, 8]. Our case is unique in that it is the first reported case of isolated endogenous endophthalmitis caused by *N. farcinica* in a patient without a clear systemic source, in the setting of immunosuppression from steroid use.

Previous reports have described poor outcomes of nocardial infections that often resulted in enucleation and death from extraocular foci [4–6]. In fact, this is the first report of isolated endogenous *Nocardia* endophthalmitis within the United States that has not resulted in enucleation [7]. In many of these cases, delays in treatment due to initial misdiagnosis contributed to the morbidity and mortality [4, 6]. Indeed the diagnosis of *Nocardia* can often be difficult given its nonpathognomonic clinical features and slow growth on culture [1, 6]. As such, it is important that *Nocardia* be considered in the differential diagnosis in any immunosuppressed patient, including those receiving steroids, who presents with signs of intraocular infection.
